# Spatial auditory presentation of a partner’s presence induces the social Simon effect

**DOI:** 10.1038/s41598-022-09628-5

**Published:** 2022-04-04

**Authors:** Arina Kiridoshi, Makoto Otani, Wataru Teramoto

**Affiliations:** 1 Graduate School of Engineering, Kyoto University, Kyoto, 615-8540 Japan; 2 Department of Psychology, Kumamoto University, Kumamoto, 860-8555 Japan

**Keywords:** Attention, Cooperation, Human behaviour, Engineering

## Abstract

Social presence is crucial for smooth communications in virtual reality (VR). Current telecommunication systems rarely submit spatial auditory information originating from remote people. However, such information may enhance social presence in VR. In this study, we constructed a dynamic binaural synthesis system and investigated the effect of spatial auditory information of a remote partner on a participant’s behavior using the social Simon effect (SSE). The SSE is a spatial stimulus–response compatibility effect between two persons. The SSE occurs when one perceives that their partner is present. Several studies have confirmed the SSE in actual environments. We presented partner sounds diotically (i.e., without spatial information) to one group or binaurally (i.e., with spatial information) to another group through headphones without providing visual information about the partner. The results showed that the SSE was induced only in the binaural group in the current auditory VR (Experiment 1), whereas both groups exhibited the SSE in an actual environment (Experiment 2). These results suggest that the auditory spatial information of remote people is sufficient to induce the SSE and has a potential to enhance social presence.

## Introduction

Advances in information and communication technologies have accelerated the development of telecommunication systems. However, smooth communications and intellectual collaborations among users remain challenging. One reason for deteriorated communications in virtual reality (VR) is the shortage of social presence. Social presence is defined as “a psychological state in which virtual social actors are experienced as actual social actors in either sensory or nonsensory ways”^1^. From acoustical and auditory viewpoints, one reason may be the absence of other people’s positional information in the audio signals presented to the listener.

Currently, remote audio-visual communication systems employ either headphones or one or two loudspeakers to present monaural or stereophonic audio signals to a listener. Speech signals are given so that the listener localizes the sound images of multiple speakers in the same location, which reduces the so-called “cocktail party effect”^2^ and leads to listening difficulties because the listener cannot discriminate the locations of multiple speakers without inter-speaker variations in binaural cues, including interaural time differences (ITDs) and interaural level differences (ILDs), and monaural cues (spectral cues)^3^. Currently, telecommunication systems transmit all sounds originating from other people without spatial information. Social presence is a concept emphasizing the location of others. By definition, auditory spatial information originating from other people is assumed to play an important role. Indeed, Kobayashi et al.^4^ demonstrated that the effects of spatialized sounds from other people can affect listeners’ VR experience. They defined the experience as a sense of presence but not social presence. In their study, the 3D spatialized sounds of other people more strongly induced listener’s subjective experience of another’s presence in auditory VR and their physiological responses than non-spatialized sounds. However, it remains unclear whether spatial auditory information of other people is sufficient to change listeners’ online behavioral responses. Therefore, this study investigates this issue using the social Simon effect (SSE)^5^.

**Figure 1:**
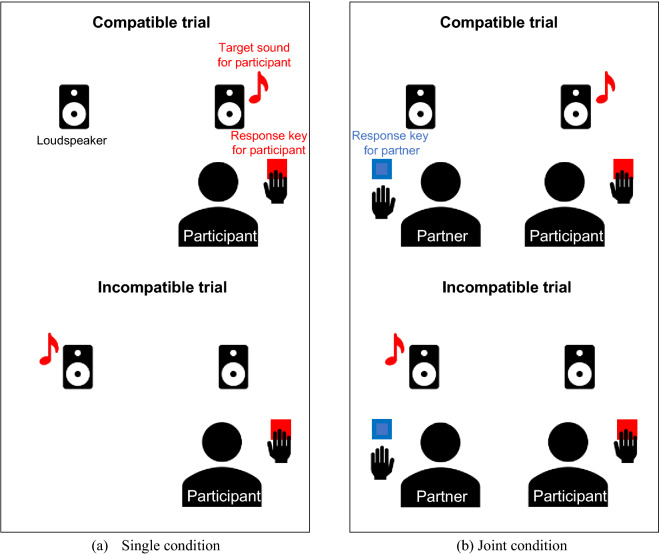
(**a**) Simple auditory go/no-go task (single condition) and (**b**) auditory social Simon effect (SSE) task with a partner (Joint condition).

The Simon effect (SE) refers to a phenomenon where the compatibility of spatial positions of a stimulus and a response key (spatial compatibility) affects a participant’s behavior^6,7^. To evaluate auditory SE, typically participants press a left or right key in response to non-spatially defined auditory attributes randomly presented on the left or right side. Responses tend to be faster when the target sound and key are spatially congruent (compatible) compared to spatially incongruent (incompatible). The SSE^3^ is the SE that occurs between two persons. In the auditory SSE, a participant responds to one type of target sound with either key, while a partner sitting beside them responds to another type of target sound with a different key. Without a partner (Fig. 1a: single), the SE does not appear because it is a simple go/no-go task. However, the participants’ responses are faster in the compatible trials than in the incompatible ones when a partner is sitting beside the participant (Fig. 1b: joint), which is also observed in the SE.

Sebanz et al.^5^ and Tsai et al.^8^ argued that the SSE is induced because a participant represents their partner’s task as their own (co-representation). Namely, a stimulus presented in a space where the partner is present activates the participant’s representation of the partner’s response. Because this conflicts with a response to be made by the participant, it affects the participant’s own response (See Dolk et al.^9^ for a different interpretation of the underlying mechanism; we will discuss this in the Discussion section).

Accordingly, the SSE should be induced in VR environments if the presence of another person is to be properly represented by the user. Suzuki et al.^10^ investigated whether the SSE and the event-related potential (ERP) can be measures for social presence in VR environments. The partner’s movements were tracked and drawn as a wireframe avatar. The influence of prior communication between a participant and their partner was also manipulated. They confirmed that the SSE is induced in VR as well as an actual environment if the participant observed the partner’s movement regardless of prior communication. By contrast, the ERP component was observed only in the actual environment or in the VR environment with prior communication. These results suggest that SSE and its related ERP component can be measures to evaluate social presence, but are associated with different aspects of social presence in the VR environment.

To clarify the effects of auditory information regarding the partner’s presence on social presence, this study investigates two aspects. The first is whether the SSE is induced by auditory cues about the partner’s presence. The second is whether the spatial information involved in the sounds originating from the partner (partner sounds) and the partner’s key-pressing sounds (response sounds) affects the SSE. This study employs auditory SSE tasks because Lien et al.^11^ and Puffe et al.^12^ reported that the correspondence between the modalities of the stimulus and the cues regarding the partner’s presence affects the induction of SSE. The experimental system utilizes a dynamic binaural synthesis, which enables an auditory stimulus presentation with controllable auditory spatial information (see the next section for details), whereas conventional auditory SSE tasks generally use a pair of loudspeakers for stimulus presentation. In this study, two psychological experiments were performed to explore whether the SSE is induced in the auditory SSE tasks when the partner and response sounds are presented with and without spatial information of the partner’s location. That is, sounds are presented diotically (identical monaural signals to both ears without spatial information) and binaurally (with spatial information) through a set of headphones.

## Methods

### Dynamic binaural synthesis system

**Figure 2:**
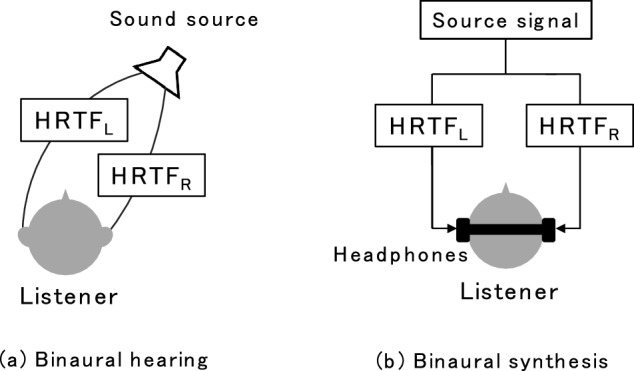
(**a**) Binaural hearing of a sound source. (**b**) Virtual sound image presentation by binaural synthesis via signal processing with a source signal and head-related transfer functions (HRTFs).

Binaural reproduction controls sound signals at both ears of the listener using headphones so that the signals are identical to those observed in a primary acoustic field. Thereby, the listener experiences the same spatial auditory space as in the primary acoustic field. Binaural signals include room characteristics such as reflected sounds and acoustic characteristics produced by a human body. The acoustical effects of the human body on binaural signals are called head-related transfer functions (HRTFs). HRTFs include binaural cues such as ITDs and ILDs, which are important for sound image localization in the horizontal plane, and monaural cues (spectral cues), which are necessary for localization in the median or sagittal plane^2^. Binaural signals naturally include HRTFs when a listener is in a primary acoustic field. In an acoustic field with a listener and a single sound source, the binaural signals observed at both ears for a given sound source are expressed as time-domain convolutions of the source signal radiated from the sound source with the HRTFs at the left and right ears for the given sound source position relative to the listener’s position (Fig. 2a, respectively labeled as HRTF_L_ and HRTF_R_). If such HRTFs are available as finite impulse response filters, binaural signals can be computationally synthesized from a sound source signal and HRTFs (Fig. 2b, binaural synthesis). This enables flexible creation of spatial auditory scenes.

**Figure 3:**
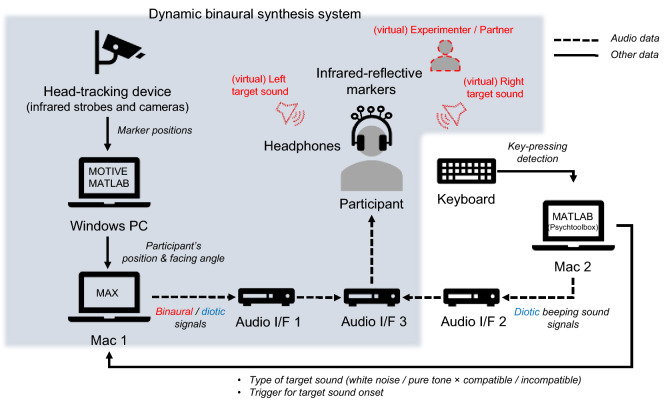
Schematic illustration of the dynamic binaural synthesis system for the auditory social Simon effect (SSE) tasks. Gray indicates a dynamic binaural synthesis system. In a binaural presentation, virtual sound images of target sounds (white noise or pure tone), experimenter’s instruction speech, partner sounds, and response sounds are presented so that they are localized by the participant at the given positions. By contrast, in a diotic presentation, these sound images, except for the target sounds, are presented diotically to both ears of the participant without binaural synthesis processing, causing in-head localization.

A dynamic binaural synthesis system spatially presented auditory stimuli (i.e., left and right target sounds for the SSE tasks, the partner sounds, and the response sounds) (Fig. 3). A non-contact head tracking device equipped with infrared strobes, cameras, and infrared-reflective markers installed on the headphones’ headband detects the participant’s head movement. Because HRTFs are switched in real time in response to the participant’s facing angle and head position, the system presents appropriate binaural signals and a physically valid auditory space, even when the participant’s head moves. The head tracking device (V120 Duo, OptiTrack) detects the participant’s head motion at a 120-Hz sampling rate. The acquired data (head position and rotation) are sent from the tracking software (MOTIVE, OptiTrack) and MATLAB (Mathworks) operating on a Windows PC (ProBook 430 G5, HP) to an audio programming environment (MAX, Cycling ’74) operating on Mac 1 (MacBook Air, Apple) as OSC (Open Sound Control)^13^ messages. Five infrared-reflective markers are installed on the headphone’s headband. Infrared cameras detect the positions of the markers. In MOTIVE, a spherical body is generated from the markers’ positions, where the origin is the center of the participant’s head. Detected motion data of the participant’s head are converted to a quaternion in MATLAB before sending to MAX. In MAX, the positions of virtual sound sources relative to the center of the participant’s head are calculated and appropriate HRTFs are selected from a database. The database consists of HRTFs for sound sources located 1 m from the center of the head in 5° intervals for both the azimuth and elevation.

The HRTFs were numerically computed using the boundary element method^14^ along with a computer model of a dummy head’s torso and head (KEMAR, G.R.A.S). Although frequency characteristics of HRTFs depend on the source distance less than 1 m^15^, this study employed HRTFs for a fixed source distance of 1 m. The distance decay inversely proportional to the distance reflects the distance between the participant’s head and virtual sound sources. The selected HRTFs are convolved with respective source signals to generate binaural signals. The generated binaural signals are output from the headphones (HD598, SENNHEISER) via audio interfaces (Audio I/F 1, DUO-CAPTURE, Roland; Audio I/F 3, OCTA-CAPTURE, Roland). For diotic presentations, identical monaural source signals not convolved with the HRTFs are output to both the left and right channels of the headphones. All audio signals are processed with a 44.1-kHz sampling rate with 16-bit quantization.

### Experiment 1: Virtual partner

#### Experimental design

The experiment had three factors: task type (Single and Joint), spatial compatibility (Compatible and Incompatible), and auditory presentation (Diotic and Binaural). Task type and compatibility were within-participant factors, while auditory presentation was a between-participant factor. In the Single condition, the partner sounds and response sounds were not presented. In the Joint condition, a virtual partner was presented using auditory cues: partner sounds and response sounds. The partner sounds included non-speech sounds such as chair-squealing and cloth-rustling sounds, while the response sounds were the sounds caused by a partner’s key response. These sounds were presented diotically to half of the participants (Group A, Diotic condition) and binaurally for the other half (Group B, Binaural condition). Although these sounds were localized in the participant’s head in Group A, they were localized externally on the left side in Group B. Participants in Group B felt as if the partner was sitting next to them.

In each trial, a 300-Hz pure tone (PT) or white noise (WN) was presented as a target sound in either the right or left direction, which the participants had to discriminate. The target sound was presented binaurally, and it was perceived as if it radiated from 50-cm away. The participants were asked to ignore PT, but to press the response key as fast as possible when they listened to WN. WN was presented from the right direction for Compatible trials, but from the left for Incompatible trials.

#### Participants

The participants were 16 undergraduate and graduate students. All were right-handed and between 22 and 25 years of age (7 women and 9 men, mean age: 23.7 ± 1.20 [standard deviation] years) with no history of hearing problems. After providing informed consent, they were randomly divided into two equal groups (Group A and Group B). All participants were unaware of the experiment’s purpose. The study was approved by the Ethics Committee of the Graduate School of Engineering, Kyoto University and performed in accordance with the principles of the Declaration of Helsinki.

#### Experimental setup

The experiments were performed using MATLAB with the Psychophysics Toolbox extensions (Psychtoolbox)^16–18^ implemented with a dynamic binaural synthesis system (Fig. 3). Psychtoolbox operating on Mac 2 (MacBook Air, Apple) controlled experimental parameters such as randomly selecting the condition, stimulus onsets, and response acquisition. Mac 2 sent the parameter data regarding the target sound (type: white noise or pure tone; position: left or right) for the SSE and the presence of partner to MAX on Mac 1 as OSC messages. Then, Mac 1 created virtual sound images at the given positions through the dynamic binaural synthesis system. Additionally, Mac 2 delivered beeping sounds to indicate the start and end of each session via an Audio I/F 2 (DUO-CAPTURE, Roland), without going through Mac 1. The audio signals from Macs 1 and 2 were mixed through Audio I/F 3 and subsequently delivered to the headphones worn by the participant.

The experiment was conducted in a sound-proof room at Kyoto University. Two sets of tables and chairs were placed side by side in the room. The set on the right side was for the participant, while that on the left was for the partner. The set on the left was not necessary, but it was placed to reinforce the participant’s belief of acting with the partner. In the Joint condition, an opaque partition was set between the tables to eliminate visual cues regarding the partner’s presence.

#### Stimuli

**Figure 4:**
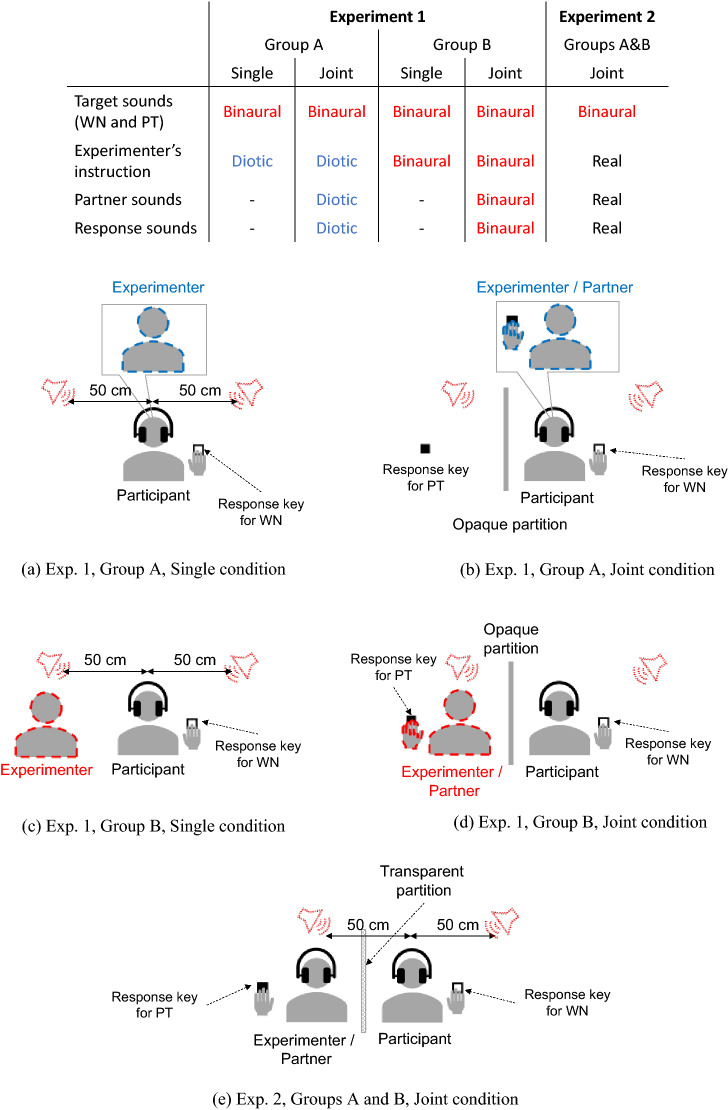
Schematic views of (**a**) Single and (**b**) Joint conditions of Group A in Experiment 1, (**c**) Single and (**d**) Joint conditions of Group B in Experiment 1, and (**e**) Groups A and B (Joint condition only) in Experiment 2. Matrix at the top summarizes the audio conditions. In Experiment 1, target sounds, white noise (WN) and pure tone (PT), were presented binaurally to Group A but the experimenter’s instruction was presented diotically. Partner and response sounds were absent in the Single condition and presented diotically in the Joint condition. In Experiment 1, target sounds and experimenter’s instruction were presented binaurally to Group B. Partner and response sounds were absent in the Single condition and presented binaurally in the Joint condition. Opaque partition was set between the participant and the virtual partner in the Joint condition in Experiment 1. In Experiment 2, an actual partner sat beside the participant. Only target sounds were presented binaurally to both the participant and the partner. Transparent partition was set between the participant and the partner.

There were four types of auditory stimuli (Fig. 4): target sounds, experimenter’s instruction, partner sounds, and response sounds. The target sounds, WN and PT, had durations of 300 ms. Their amplitudes were adjusted so that the sound pressure levels (A-weighted) were respectively 70 dB and 65 dB at the participant’s left ear when presented from a left virtual sound source through the headphones, as measured by a head and torso simulator (4128C, Brüel & Kjær). Sounds were binaurally presented as either a left or right virtual sound image located 50 cm from the center of the participant’s head.

The experimenter’s instruction speech for the procedure of the experiment, the partner sounds, and response sounds were recorded in prior to the experiments in a sound-proof room. The amplitudes of the partner sounds and response sounds were adjusted so that they sounded as loud as the actual ones in the preliminary experiments, resulting in a maximum of 65 dB and 50 dB of sound pressure level at the participant’s left ear, respectively. One session of the auditory SSE tasks took approximately 11 min. Thus, the partner sounds were recorded for more than 11 min to ensure that they were presented to the participant throughout the session. The partner sounds were not synchronized with WN or PT. Therefore, it was possible that the partner sounds were presented at the same time as WN or PT. Hence, the partner sounds may have interfered with WN or PT, which might affect the RTs. However, treatment to avoid interference was not made because such interference may also occur with an actual partner. The response sounds were delayed for 400 ms from the PT presentation.

Additionally, the diotic beeping sounds signaling the onset and offset of each session were created by a built-in function of Psychtoolbox (600-Hz pure tone, 1-s duration).

#### Procedure

In the Single condition for Group A (Fig. 4a), the participant performed the trials without listening to the partner or response sounds. The experimenter’s instruction speech was presented diotically to the participant through the headphones at the beginning of the session. Two seconds after the instruction speech ended, a diotic beeping sound indicated the start of the session. The first trial began two seconds after the beep. The trial ended when the participant responded or 1 s after the target presentation. The next trial started 1 s later. After the 240^th^ trial, a diotic beeping sound indicated the end of the session.

In the Joint condition for Group A (Fig. 4b), the participant performed trials while listening to diotic partner sounds and response sounds. After the participant was seated on the chair, the experimenter placed a response key and a chair for the partner so that the participant recognized the partner would use them. For the WN trials, where the participants needed to press the response key, the trial ended either when the participant responded or did not respond within 1 s. For the PT trials, where the virtual partner was supposed to press the response key, the diotic response sound was presented to the participant 0.4 s after the onset of PT. If the participant mistakenly responded within 0.4 s, the trial ended soon after the partner’s response without presenting the response sounds. The other procedures were the same as in the Single condition for group A.

In the Single condition for Group B (Fig. 4c), the participant performed the trials without listening to the partner or response sounds. The experimenter’s instruction speech was presented binaurally, as if the experimenter spoke on the left side of the participant, 50-cm from the center of their head. The other procedures were the same as the Single condition for Group A. In the Joint condition for Group B (Fig. 4d), the participant performed the trials while listening to the binaural partner sounds and response sounds, the binaural experimenter’s instruction speech, and the binaural target sounds. The other procedures were the same as the Joint condition for Group A.

It should be noted that non-spatialized (diotic) or spatialized (binaural) presentation of instruction speech may affect the participants’ responses in the SSE tasks. Therefore, the experimenter’s instruction speech was consistent with the partner and response sounds and presented diotically or binaurally for Group A or B, respectively. This eliminated possible effects of spatial/non-spatial presentation of the instruction speech.

The participants were asked to ignore PT, but to press the response key as fast as possible when they heard WN. One session was assigned to the Single condition and another to the Joint condition. Each session consisted of 240 trials (spatial compatibility (Compatible/Incompatible) × target sound (WN/PT) × 60 repetitions). There was a 10-min break between the sessions. The condition presentation order was counterbalanced in each group. The participants were asked to close their eyes during the sessions and to press a response key on the keyboard with their right hand. The participants were also asked to face forward as much as possible while listening to WN or PT, but they were not forced to keep their head still throughout the session. Prior to the start of each session, there was a short practice session of 48 trials to familiarize the participants with the procedure. After the participant finished the practice session, the experimenter left the room, and the participant started the session by oneself.

### Statistical analyses

Trials where RTs exceeded the lower limit (150 ms) or upper limit (1,000 ms) were excluded from the following analyses as outliers. In each group, participants generated more than 48 effective trials (80% of 60 trials) in all the conditions. Therefore, all trials, excluding outliers, were analyzed. A median value of RT was used as a representative value for each participant. The normality test (Shapiro–Wilk test) revealed the mean RTs were normally distributed in all of the conditions (*p* > 0.05). Thus, two-way repeated-measures analysis of variance (ANOVAs) was applied to median RTs with the within-participant factors of task type (Single/Joint) and compatibility (Compatible/Incompatible). The simple main effects were tested for interactions identified as significant (*p* < 0.05) in the two-way repeated-measures ANOVA. The same analysis was applied to the outlier rates and error rates. Furthermore, to quantitatively evaluate the effect of compatibility including null effects, we also analyzed the RT data using a Bayesian approach. Specifically, we performed the Bayesian paired sample *t* tests and calculated the Bayes factor (BF_10_). The BF_10_ values were interpreted based on the classification scheme proposed by Jeffreys^19^.

### Experiment 2: Real partner

Experiment 1 showed that the SSE was induced only in the Joint condition for Group B. The SSE did not appear in the Single condition for Group B or in any condition for Group A. However, this might reflect the participants’ susceptibility to the SSE. Thus, Experiment 2 was performed with the same participants as Experiment 1 to investigate whether the SSE is induced in a real environment when a partner actually sat beside the participant and performed the tasks with the participant. The auditory SSE tasks were performed with the same target sounds (WN and PT) as in Experiment 1. The experimental system itself did not present the partner sounds, response sounds, or experimenter sounds. Instead, an actual experimenter and partner sat beside the participant, respectively to provide instruction and perform the tasks with the participant.

Only the Joint condition was performed. Figure 4e illustrates Experiment 2. A keyboard, including a response key for PT, and a chair for the actual partner were placed on the participant’s left prior to the session. The experimenter also served as the partner. The partner listened to the same auditory stimuli as the participant by wearing another set of headphones. A transparent partition was placed between the participant and partner. Once the participant and partner were seated, the participant started the session after the beeping sound, which was presented after the experimenter’s oral instruction to indicate the start of the session. The experimenter’s instruction provided the same information as Experiment 1. The first target sound was presented 2 s after the beeping sound. For WN trials, the trial ended either when the participant responded or did not respond within 1 s. For PT trials, the trial ended soon after the participant mistakenly responded within 0.6 s. Otherwise, the trial ended 0.6 s after PT onset regardless of the partner’s response. This was due to a limitation of the experimental system because it could only recognize one keyboard response per trial. It should be noted that, however, the partner responded to all PTs within 0.6 s in all sessions. The next trial started 1 s after the previous trial ended. After 240 trials, a beeping sound indicated the end of the session.

The normality test (Shapiro–Wilk test) revealed that the mean RTs were normally distributed in all conditions (*p* > 0.05). Paired *t*-tests with a factor of compatibility (Compatible/Incompatible) were applied to median RTs. The results of Experiment 2 were analyzed in two groups (A and B) separately to assess the inter-participant, or inter-group, variations in SSE inducibility under identical conditions. The outlier rates and error rates were also analyzed in the same way. We performed the Bayesian paired sample *t* tests to evaluate the effect of compatibility on the RT data including null effects.

## Results

### Experiment 1: Virtual partner

**Figure 5:**
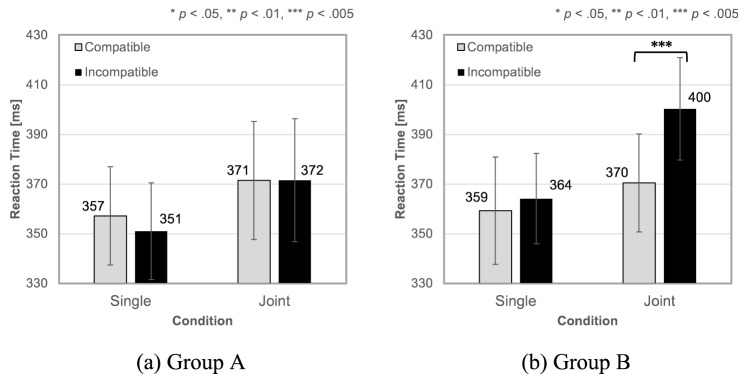
Mean Experiment 1 reaction times (RTs) for (**a**) group A (diotic, *n* = 8) and (**b**) group B (binaural, *n* = 8) under Single and Joint conditions. Error bars indicate standard errors of the means.

Figure 5a illustrates the mean RTs for compatible and incompatible trials in the Single and Joint conditions of Group A (diotic sound). The two-way repeated-measures ANOVA revealed no significant main effects or interactions. The Bayesian paired t-tests with a factor of compatibility resulted in BF_10_ = 0.709 for the Single condition and BF_10_ = 0.336 for the Joint condition. Thus, diotic partner and response sounds did not induce the SSE. Table 1(a) shows the mean outlier rates and mean error rates for compatible and incompatible trials in the Single and Joint conditions of Group A. The error rate in each condition was less than 1.46%. The two-way repeated-measures ANOVA on the outlier and error data revealed no significant main effects or interactions. Thus, the difference in error rates (i.e., speed-accuracy tradeoff) cannot account for the absence of SSE.

**Table 1:** Mean outlier and error rates in the Single and Joint conditions for (a) Group A (diotic sound) and (b) Group B (binaural sound), Experiment 1. Significant main effects or interactions are not observed.

	Single		Joint	
	Compatible	Incompatible	Compatible	Incompatible
**(a) Group A**				
Mean outlier rate [%]	0.21	0.21	1.04	0.21
(SD)	(0.55)	(0.55)	(1.16)	(0.55)
Mean error rate [%]	1.04	1.46	0.63	0.63
(SD)	(1.43)	(2.11)	(1.16)	(0.81)
**(b) Group B**				
Mean outlier rate [%]	0.42	0.63	0.42	0.21
(SD)	(1.10)	(1.16)	(0.72)	(0.55)
Mean error rate [%]	0.83	1.46	1.04	0.42
(SD)	(1.18)	(2.11)	(0.81)	(0.72)

Figure 5b illustrates the mean RTs for the compatible and incompatible trials in the Single and Joint conditions of Group B (binaural sound). The two-way repeated-measures ANOVA revealed significant main effects of condition (*F*_1,7_ = 19.08, *p* = 0.003, *η*_*G*_^2^ = 0.732) and spatial compatibility (*F*_1,7_ = 18.07, *p* = 0.004, *η*_*G*_^2^ = 0.721), and a significant interaction effect (*F*_1,7_ = 18.24, *p* = 0.004, *η*_*G*_^2^ = 0.723). Simple main effect tests revealed that the mean RT was significantly shorter (*F*_1,7_ = 45.97, *p* < 0.001, *η*_*G*_^2^ = 0.868) in compatible trials (mean ± standard deviation: 370 ± 59 ms) than in incompatible trials (400 ± 65 ms) only in the Joint condition. The Bayesian paired sample *t*-tests with a factor of compatibility resulted in BF_10_ = 0.455 for the Single condition and BF_10_ = 125.004 for the Joint condition. Thus, the binaural partner and response sounds induced the SSE. Table 1(b) shows the mean outlier rates and mean error rates for compatible and incompatible trials in the Single and Joint conditions of Group B.

The error rate in each condition was less than 1.46%. The two-way repeated-measures ANOVA on the outlier and error data revealed no significant main effects or interactions. Consequently, the difference in error rates (i.e., speed–accuracy tradeoff) cannot fully account for the observed SSE.

### Experiment 2: Real partner

**Figure 6:**
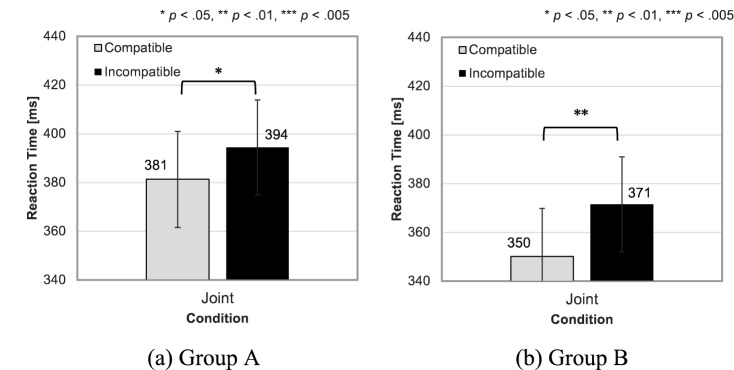
Mean Experiment 2 reaction times (RTs) for (**a**) Group A (*n* = 8) and (**b**) Group B (*n* = 8) under the Joint condition with an actual partner. Error bars indicate standard errors of the means.

Figure 6 illustrates the mean RTs for compatible and incompatible trials in the Joint condition of Groups A and B. Two separate paired *t*-tests on each group’s RT data revealed a significant effect of compatibility in both groups (Group A: *t*_7_ = 2.46, *p* = 0.043, *d* = 0.291; Group B: *t*_7_ = 3.54, *p* = 0.010, *d* = 0.394). The mean RT was shorter in the compatible trials (Group A: 381 ± 44 ms; Group B: 350 ± 54 ms) than that in the incompatible trials (Group A: 394 ± 46 ms; Group B: 371 ± 54 ms). The SSE was induced irrespective of the group when the partner was physically next to the participant. Table 2 shows the mean outlier rates and mean error rates for the compatible and incompatible trials in the Joint condition of Groups A and B. The paired *t*-tests revealed that neither group showed a significant difference in the mean outlier rates between the compatible and incompatible trials. As for the error rate data, the paired *t*-tests revealed no difference between the compatible and incompatible trials in Group A. However, a significant difference was observed in Group B (*t*_7_ = 2.65, *p* = 0.033, *d* = 0.676). In Group B, the mean error rate was larger in the compatible trials (1.46 ± 1.30%) than that in the incompatible trials (0.63 ± 1.16%). Hence, the difference in error rates cannot fully explain the difference in RT.

**Table 2:** Mean outlier and error rates in the Joint condition of Groups A and B in Experiment 2.

	Group A		Group B	
	Compatible	Incompatible	Compatible	Incompatible
Mean outlier rate [%]	0.00	0.00	0.21	0.42
(SD)	(0.00)	(0.00)	(0.55)	(0.72)
Mean error rate [%]	1.67	0.83	1.46	0.63
(SD)	(2.20)	(1.18)	(1.30)	(1.16)

Neither group shows significant differences in the mean outlier rates between compatible and incompatible trials. In Group A, the mean error rates between compatible and incompatible trials do not differ significantly, whereas that in Group B is significantly greater in the compatible trials than in the incompatible trials.

## Discussion

In Experiment 1, the SSE was induced when the partner and response sounds of a virtual partner were binaurally presented (Group B). However, the SSE was not induced when the sounds were diotically presented (Group A). This difference between groups was not attributed to the group differences in susceptibility to SSE because both groups exhibited the SSE when a partner sat beside the participant in the actual environment (Experiment 2). Thus, what matters is whether auditory information of other people is spatialized.

Several studies have investigated the effects of spatialized sounds on VR experiences. Hendrix and Barfield^20^ added spatialized (and non-spatialized) sounds to a visually simulated virtual world, which was navigated using a computer mouse. The sound sources were radio broadcasts delivering rock music and operation sounds from a soda vending machine. The results of questionnaires showed that the spatialized sound increased a sense of presence, the fidelity of users’ interaction with the sound sources, and the sense that sounds were emanating from specific locations. Västfjäll^21^ investigated whether the number of audio channels in a reproduction system affected the sense of presence, emotion induction, and emotion recognition when participants listened to music in a virtual environment. Questionnaires showed that six-channel reproduction received the highest rating of presence and emotional realism, although the effect of emotion induction was the same between stereo and six-channel reproduction.

As for research on social presence, Kobayashi et al.^4^ presented the sounds of approaching people (seven men clapping hands and a man playing a guitar) through a 96-channel sound reproduction system. They measured the listener’s subjective experience of someone’s being there using questionnaires and physiological responses of the sympathetic nervous system such as heart rate, blood volume pulse amplitude, and skin conductance level. Compared with non-spatialized sounds, the 3D spatialized sounds heightened the sense of presence of other people and induced a higher activation of the sympathetic nervous system.

These studies suggest the importance of spatialized sounds on users’ experiences in VR environments, including social presence. In addition to subjective and physiological response levels^4^, this study provides new evidence that spatialized sounds can also influence users’ experience of social presence at a behavioral level. Spatialized sounds made it possible for users in the VR to behave in the same way when they were in the actual place.

We used the SSE as a behavioral measure of social presence in VR because previous studies have indicated that the SSE occurs due to one’s automatic representation of the partner’s task or the partner themself as one’s own (co-representation account)^5,22^. This social account can well explain the behavioral findings that the SSE hardly occurred when the actor and partner had a bad mood^23^ or the partner was an out-group member^24^. However, several studies have demonstrated that the SSE can be induced when the partner does not actually coexist. For example, Tsai et al.^8^ showed that the SSE occurred if the participants believed that a partner was next to them and they performed the task together. In their study, participants were not only given instructions to insinuate that a partner was present in another room, but they actually met and did practice trials together. On the other hand, it should be noted that Sellaro et al.^25^ showed that the mere belief was not enough to induce the SSE. They found that positional information of the partner needed to be presented. Given this factor, our dynamic binaural synthesis system provided sufficient auditory information about the partner for the participants to experience a spatialized virtual partner.

There is an alternative account for the SSE. Dolk et al.^9,26,27^ proposed the referential coding account, arguing that the presence of the human or biological (or biologically-inspired) agent was not necessary. Instead, any event representation salient enough to create conflict with the participant’s relevant response could induce the SSE. For example, Dolk et al.^9^ reported that the SSE was induced by a nonliving object, which was located next to the participant and attracted the participant’s attention by sounds or movement, such as a Japanese waving cat with a mechanical moving arm and a clock with a rotating element. According to this account, it can be considered that the SSE was induced in this study because the participants experienced a spatially salient event (but not necessarily a human one) in VR by the binaural partner sounds. Nevertheless, it is noteworthy that Dolk et al.^27^ added another important assumption to the referential coding account to comprehensively explain the results of the SSE studies. Specifically, the saliency of other-generated event can be modulated by the similarity between self and the event: “Increasing the degree of similarity increases the demand of discriminating alternative event-representations”^27^, leading to larger SSE. For this reason, the SSE was hardly induced or weak when the actor and partner had a bad mood^23^, the partner was an out-group member^24^ or the partner was a non-biological agent^28^. Considering this assumption, it might be that the SSE was induced by simply presenting the binaural partner sounds in this study because the participants experienced social similarity between self and the partner, thus, social presence, by the spatialized sounds. Future studies should provide more convincing evidence for social presence by spatialized sounds in VR using subjective evaluation methods and other behavioral measures.

In conclusion, spatial auditory information of another person can play an essential role in experiencing social presence in auditory VR environments without visual cues. This implies that remote communication systems presenting monaural or stereophonic audio signals cannot induce social presence among users. However, presenting sounds originating from other people with appropriate spatial information through binaural synthesis or other spatial audio reproduction techniques based on theories of sound field reproduction^29,30^ may facilitate social presence among users in auditory VR environments or remote communication systems.

## Data Availability

The datasets generated and analyzed in this study are available from the corresponding author on reasonable request.
